# Conference on Problems Relating to the Area of Advance

**Published:** 1919

**Authors:** 


VOL. II, No. 7 PARIS FEBRUARY-MARCH 1919

### WAR MEDICINE

Published by the American Red Cross Society in France
for the
Medical Officers of the American Expeditionary Forces

### RESEARCH SOCIETY REPORTS

THE ELEVENTH SESSION OF THE RESEARCH SOCIETY OF THE AMERICAN RED CROSS IN FRANCE

November 22 and 23, 1918, at the Hotel Continental, Paris.

### CONFERENCE ON PROBLEMS RELATING TO THE AREA OF ADVANCE

GENERAL FINNEY. This meeting was planned some two months ago, at a time when we were in the midst of great activities. Today, fortunately, the war is over, but I hope we will find enough to interest us in considering some of the points that were under discussion during that period. I am glad distinguished representatives of the British, French and Italian Medical Corps are here. I am sure they will be willing to give us the benefit of their wide experience.

The transactions of this meeting will be kept in some permanent form, so that in the event of another war — which God forbid — we will have at any rate a record of the combined experiences of those who are present this morning, composing a scientific organization such as this.

Members of the Committee visited a number of hospitals to ascertain what problems they had met in their work, and what questions had specially troubled them, upon which they would like to have the opinion of others. These questions voice the thoughts and problems of many of the hospitals and medical men of the army.

As clinicians we have primarily to deal with the patient, but many problems involving the care of the patient are inseparable from those of administration. Our questions this morning largely concern the latter, and necessarily, therefore, administrators are in a position to give the best information.

DIVISION. — A. **What should be the function of the Battalion Aid Station?**

B. **Is it desirable to continue the Regimental Aid Station?**

It will be well to consider these two questions together.

LIEUT.-COLONEL TURCK. One thing that we have to consider is that it is very unfortunate that our army has not had more open warfare, because we are inclined to consider the battalion aid post and the regimental aid station from the standpoint of a fixed division, where it is possible to have all the equipment necessary, to provide plenty of litters, plenty of blankets and plenty of hot water. But the one thing we have worked on, and which necessarily will have to be worked out in open warfare, is: — What should the medical officers do in a rapidly advancing area?

When a division is advancing, no aid post is possible. When troops are going forward, the only thing for the battalion aid men to do is to carry their equipment on their backs, to give morphia, apply first aid, and go on further. It is impossible to get through shell fire, machine-gun fire, and gas, to install an aid station, except in a shell-hole or a captured dug-out; you can't take your equipment with you; it is an impossibility. There is a possibility of using Japanese push-carts, and in a rapidly advancing division that is all the men can do practically. I will read you something based on the scheme of Colonel Gressinger. . . . . . . . . . . .

. . . . . . . . . . . . . . . . . . . . . . . . . . . .

The fixed station is very little trouble to handle, but when troops are advancing it is simply a question of first aid, giving morphine, putting on dressings, etc., and going on with the troops. Also, the men will be divided; you will perhaps find two wounded men of each company, and the battalion aid officer must attend to whichever is suffering most. Under shell-fire you cannot stop to put on splints. In talking with Colonel Gressinger we thought that we could perhaps use a splinting party from the ambulance, — collect the men as well as possible and send on a splinting party with splints and blankets, and supplement the pack roll with supplies sent up by ambulance, though frequently there are no roads. Then most of the splinting could be done along with the litter section of the ambulance.

I have just one word to add about the regimental aid station. I certainly think it is a mistake to have a regimental aid station because of the number of men. More can be done if the men can be taken to a central station, but very frequently they cannot; they will stay in shell-holes. I may mention one instance, in the Argonne, when our troops were advancing and at times retiring. The regimental medical officer took care of the wounded in shell-holes for 36 hours, and he carried them back after crawling from one shell-hole to another. When organizing an aid station we do not consider all these things, and we do not always realize that it will be impossible at times to get the men there.

Colonel Pearson. We have been with the 5th Division, and during the summer we operated first in the Vosges, then at St. Mihiel, then north of Verdun, and lastly across the Meuse. In each part we had entirely different positions, and in each plan of attack the position of the troops, the artillery, and the machine-guns, were entirely different, and the scheme of evacuation of wounded and care of the men, the disposition of the battalion and regimental aid stations and infirmaries had to be adapted to the terrain and military situation at that time; so we cannot quote any fixed law or rule as to where the aid posts will be, what they will take care of, what will be the function of the regimental aid station, etc. If you are working with the infantry and advancing with a wide sector of the front, you may have one battalion in reserve and one for a certain part of the sector, you may have a whole division on a front of 3 kilometres, or you may have a division spread out over 30 kilometres in another place.

I want to give tribute to the men who have served as battalion surgeons; to my mind they are the most important link between the Medical Department and the patient. Our function is to take care of the troops, and it is through the regimental and battalion aid surgeons that we come in contact with our patients. These are the men who have been going with the infantry right forward, who have been sleeping out in the mud, who have suffered all the hardships that the troops have undergone in the advance, who have worked under shell-fire, under rifle fire, under machine-gun fire, and they have the admiration and respect of the men working with them. These are the men who have kept us in touch with our patients and who have given the Medical Department what standing it has with the fighting line. Our soldiers have admiration for the regimental and battalion surgeons, as they have for the ambulance section and for the hospital surgeons.

We have not had enough battalion surgeons, because some of these men have been killed. The dental surgeons assigned to the divisions have also been right out on the front doing first aid work, and several of them have been cited for conspicuous courage under fire.

The battalion aid station does not amount to much as to material; you must bring up what you can. In the St. Mihiel sector we had only one road, and that was soon blocked by heavy artillery, large caterpillar tanks and the infantry, and the regimental aid stations were pitched 25 kilometres from the rear. The men took the material up on litters. One night, during the advance, the troops had to pass through woods, and a great deal of material was lost, so that in the morning, when we went over the top, the sanitary troops of the line carried what they could in their belts and in sacks, and with what they could climbed out of the trenches and through the barbed wire. The supply officer of the division sent on splints, etc., by the first ambulance that went through.

Primary aid must very often be carried on the men's backs; with trenches to jump, barbed wire, then more trenches and more wire, it is perfectly obvious that no pack mule can get through and no medical cars either. There were no roads; all roads had to be filled in by the engineers as we advanced. If we could not use the main roads, why did we not go through the fields or by the side roads? But neither mules nor cars could go through 11 foot trenches, shell-holes, or barbed wire entanglements. The regimental aid station takes what it can carry, and is supplemented by the first ambulance to get through the lines.

In the battalion aid station, conditions are sometimes different. One battalion goes on first and lies down on the grass, then another goes through and beyond, so they go up on the same line. Then you must put the battalion aid station where you can, which makes an entirely different situation. The ambulance dressing station would be able to get some ambulances to the regimental aid station, and later in the day to the battalion aid station. Conditions have to conform and vary with the conditions of attack.

When the regimental station gets to a place where it can stay for a week or so, it requires a certain amount of records. Records of the men and of the personnel must be kept, and also form 4. You therefore need a typewriter and some office equipment. There is also the question of transporting a certain quantity of material and supplies. The form of equipment for transporting regimental and battalion supplies is quite inadequate. The best solution for that problem would be to have a Ford ambulance allowed to each battalion surgeon, which would take his material as far as it could go, and if there is no obstruction the ambulance could run to the first-aid dressing station and evacuate a few of the wounded.

With regimental infirmaries we are allowed one wagon which carries part of our supplies, but not as much as we need. There is also no provision made in the tables of organization for the transportation of supplies for the dental surgeons; there is nothing to carry their chests on. Each regiment should be given one truck to carry supplies. For the small infirmary or dressing station, I would recommend the substitution of the French tent for the present tent with short poles; the small type of tortoise tent can be carried on an ambulance and give sufficient room for the battalion needs.

Colonel Cummins. I think that we all know what ought to be done when fighting trench warfare, — that is quite simple. But a very interesting and difficult problem raised by the first speaker is as to what ought to happen during open warfare. I think that under conditions of moving warfare one should not talk so much of the location and function of the battalion aid post as of that of the attending medical officer. It is he who represents the medical aid of the station, and his place is a shifting location. One principle ought never to be forgotten, and that is that the first duty of the battalion medical officer is to make known his whereabouts. He cannot go constantly runnning after the battalion, he must locate himself and let the field ambulance and the battalion know where he is, so that he can be sent for. The battalion will break down at a certain point and have casualties; it must then be possible for the battalion commander to communicate with his battalion medical officer.

The battalion medical officer must make arrangements to get information as to where the wounded are, and then go forward and deal with them — in a shell-hole if necessary, but he must let people know where he can be found. I have had this difficulty myself and I know perfectly well that the first thing is to be located, so as to be easily found.

In regard to supplies, the field ambulance must help out the battalion medical officer. The field ambulance commander, under orders of the division surgeon, must always send ambulance detachments to co-operate with the battalion medical officer, and to bring up the field stretchers. Though these are intended to bring the wounded back, they can be used to run up dressings and other things to the place where the battalion surgeon is.

The first duty of the battalion surgeon is to try to keep in touch with the commanding officers of the field ambulance and of the battalion.

GENERAL FINNEY. C. **What should be the location, capacity and function of the Ambulance Dressing Station?**

LIEUT. SUTTON. In dealing with the ambulance question, a method which was first mentioned by a British colonel was adopted in a number of divisions in our service.

Assuming that a division would have a triage, the ambulance corps would be located wherever there was a good place and good hard standing for the ambulances, because in the forward area there is a lot of mud and the ambulances must be kept in good condition. A motor repair section is stationed a little before each road crossing, and whenever possible one is worked up as a post-battalion aid station. The system is so worked that as each ambulance comes through the rear, the patients are not changed from one ambulance to another, but as this ambulance passes the next simply goes back and takes its place. In this way you get an efficient use of the ambulances by the method of circulation, the personnel gets a chance to rest, and there is time to clean and repair the car. In some divisions especially, it is remarkable how clean the ambulances have been kept at all times.

Of course, the ambulance company has not only the side of transportation, but it has the question of ambulance dressing stations. The number of dressing stations is determined by the type of sector and type of warfare. It has been found especially satisfactory to have two dressing stations for a divisional sector, and in a rapid advance to have only one functioning at a time and the other moving forward and setting up; if possible another can be packed up and ready to go forward.

In crossing no man's land, where the line has been fixed for some time, it frequently happens that a big road is blocked, due to mining or the destruction of the rails of communication; these mines explode after a certain time, and if you can get the dressing station across before they blow up, it gives you time to care for the wounded.

LIEUT.-COLONEL TURCK. I only wish to mention one point, and that is that when the roads are entirely blocked and no ambulance can move, no ambulance scheme will work out. You must then look out for other transportation, — trucks, ammunition trains going up, etc., — anything that is going forward. You can fill up trucks that are going back; mule ambulances can be got along where others fail to pass, and, worked from the ambulance dressing stations, they have done a good deal of fine work. When the roads are blocked and the ambulance service breaks down, if there is any kind of open territory you can depend on mules and thus relieve crowding of the dressing stations. It is a matter of utilization of every bit of transport available : mules, railways, munition trains, push carts, etc.

General Finney. This question is largely one of transportation, and it will be interesting to get the experience of the British Army because their conditions were different. We had a great deal of mud, but not as much as they had.

Colonel Cummins. As I had to go through this experience I know something about transport. I think that we must retain all transport, including mule ambulances. I think the old ambulance wagon is not very good; it is much too heavy. What I would like is a little, light, two-horse ambulance. We have a light ambulance wagon that can go wherever a gun can go; it is very useful, and I think it ought to be supplied to the infantry field ambulances in the future. We must have 7 motor ambulances per field ambulance, and they have been a very good replacement for the large, heavy ambulances of past days.

I would like to see at least 3 light ambulance wagons to every division. It seems to me that you could use horses if mules are not available, but you want to get good pack saddles that will take stretchers. Stretchers are important to get forward, but they are not easy to carry. A good pack saddle has to be well thought out. I think the field ambulance of the future should have a pack transport; at any rate I am a believer in a few horse-drawn vehicles and means of utilization of pack transport when necessary.

General Finney. D. **What should be the function, location and control of Field Hospitals?**

Lieut.-Colonel Lee. I have been with the 2nd Division for 8 months and we worked out the following system : —

1st. — Hospital reserved as triage, and specialized in triage.

2nd. — Gas and medical hospital with 200 to 250 beds.

3rd. — Hospital for wounded. This is organized as a mobile hospital for seriously wounded.

In our division I think this worked out very well. The triage hospital was very far forward; the men working there were specially trained in splinting, etc.; in the medical and gas hospital we had the men of the division who were most adapted to medical work, and for the seriously wounded hospital we had trained surgeons.

The Ambulance Companies were not used as triage. The entire front line of the field was handled by an Ambulance Company. I have always felt that if the mobile field hospital is properly organized and supervised, it can get where it is wanted very quickly. From the standpoint of the troops it helps the fighting men materially to know that the same group is always waiting for their division.

General Finney. As I understand them, conditions in this war are entirely different in regard to field hospitals from conditions in preceding wars.

In our army at the present time no operating is planned to be done in the field hospital, but in exceptional circumstances it has to act as a mobile hospital.

There is a good deal of discussion at present as to the equipment of the field hospital. Should there be one or two field hospitals kept for emergency surgery? In rapidly advancing lines everyone appreciates this advantage.

It has been pointed out in this war that if you have facilities for operation, the temptation will always come for those facilities to be used when it may be unnecessary. Is it for the greatest good of the greatest number to have field hospitals equipped to do major surgical work, or to keep them entirely out of surgical work and utilize the mobile hospital attached to the division or corps? That is a very important point.

Lieut.-Colonel Clinton. My ideas are based exclusively on the fact that we have two distinct types of warfare. In a big push no scheme works perfectly, and it seems that the establishing of non-transportable hospitals or mobile hospitals is purely a matter of transportation and a matter of the number of wounded. When some hospitals have 1400 patients come in in a period of 4 hours, all they can do is triage work, and it was observed that the most satisfactory triage of patients was done in those hospitals that attempted no operative work. The first cases that came down improperly splinted came from an ambulance that started to do its own surgical work. It is quite enough for field hospitals to do triage work.

General Wallace. As General Finney has said, this is a very important point. In the British Army we started with the idea of doing operations in field hospitals, and these were equipped for that work and had surgeons. One of the wisest things our army did was to decide that no surgical operations should be done at the field ambulances, but at the casualty clearing stations. Taking all in all, I believe the greatest good is done for the greatest number by doing the operating in the casualty clearing stations and allowing the field ambulance people to do fetching and carrying work. I always tell the men that really they are doing work which is as important as that of the surgeons back in the clearing stations. Surgeons are felt to be very scarce in the army, and if they are concentrated in the casualty clearing stations they will do more work.

In stable warfare I believe in putting teams into field ambulances and making proper forward operating centres. In winter time, especially in the case of shattered limbs, I believe that a team sent to a field ambulance will do good work, but that in general it is bes' to keep surgeons in the clearing stations.

Colonel X.... In an advance movement, and especially in cold weather, the function of the field hospital is often to provide shelter and cover for the men, because if they are left out in a heavy frost over night many of them will be dead by morning. If they are sent away to the evacuation hospital the distance is often very great. One of the longest distances recorded to get men to an evacuation hospital was 75 miles. If patients are put on ambulances and carried that distance, some will be dead before getting to the hospital.

In our division, the field hospital went forward, following as closely as possible behind the troops. It was necessary as the troops advanced to push the first hospital up. It should not, however, be placed at cross roads where it is liable to be under shell-fire. We had 1200 cases evacuated during a period of 14 days. We had one operating team and one shock team, and we found that we were able to take care of 2, and in some cases 3, divisions. The only type of operating done there was in cases that would have died if they had been taken to an evacuation hospital.

The gas hospital was set up where there could be shower baths, etc. One of the most important stations for slightly wounded ceased to exist when we had triage hospitals. The slightly gassed cases should be eliminated, because intoxication by gas is a matter of degrees and many of the slightly gassed men can continue in the line. In one instance, 3500 men sent as gas cases were sent back.

It is an absolute nec ssity to push hospitals forward as quickly as possible to take care of the elements making the attack. We found that we could usually get the hospitals moved up at night. One of the first units to cross the Meuse was a field hospital, in order to provide shelter for the men who had made the attack during the day.

LIEUT.-COLONEL X.... I don't really feel that I had better say very much, as my personal experience is not sufficient. However, I feel very strongly that in field hospitals and all advanced formations every preparation should be made for the care of surgical shock. The desirability of having better preparations for the care of surgical shock in triages and everywhere else is very great, and what has been said as to the value of the field hospitals for keeping patients warm is extremely important.

LIEUT.-COL. POOLE. One feature that I feel very strongly about is that there is a great leaning towards doing operative work in field hospitals. I have seen operations done on the knee joint, on the cranium, on fractured tibias, and many other cases which could have been sent to better equipped hospitals.

Triage hospitals generally have not the personnel, the equipment, nor the facilities for hospitalization for a proper period to give patients the best chance for recovery. This applies to individual cases; the greatest good for the greatest number is very apt to be overlooked. Surgery in general should be done by pushing up some sort of small mobile unit as near as possible to the triage, where the work can be done far better than in any field hospital. Field hospitals should concentrate on the treatment of shock, and on triage.

At Mobile Hospital No. 2, it took 20 minutes for 5 medical officers to sort out from 2 trucks the seriously wounded cases which they should take. Loss of time is avoided by putting the slightly wounded in one truck and the seriously wounded in another. Efforts should be directed towards triage and treatment of shock, and operating should be done by small mobile groups pushed up as far as possible.

LIEUT.-COL. VAUGHAN. Personally I feel that as our field hospitals exist at present there is no call for them and no place. They should be kept for gas, medical, and triage work. We cannot lay down any definite rules as to what type of surgery should or should not be done in field hospitals. At our hospital we had one tent as a triage, and four other tents placed as follows : —

1. Gas and medical. 2. Walking cases. 3. Stretcher cases. 4. Slight stretcher cases.

Ambulances coming in went to the proper tent from which the patients were evacuated to proper hospitals.

But when the roads became blocked, all we had was one tent for shock cases, associated with another tent from another hospital for non-transportable cases. The work there was work that had to be done to save life.

In one instance 80 o/o of the cases were gas infection or slightly wounded cases. When this happens, our function changes. The slightly wounded should be operated and the seriously wounded left alone. The time element is really the important factor.

A small mobile surgical unit should be attached to every division in open warfare. The type of large casualty clearing station used in the French, British and Belgian armies is especially valuable in fixed warfare, but a small unit can be put up in 4 to 6 hours, and can take care of troops who would die from gas infection if there were no means of taking care of them.

Colonel Brooks. One point is very important ; everyone has spoken of the importance of proper triage of gassed and medical cases. One of the greatest faults in the triage and field hospitals is that influenza, measles and scarlet fever are distributed among gassed cases. You should separate the gassed cases, which are susceptible to infection, from infectious cases and also from the slightly wounded. This should be carefully done. The evacuation hospital is the worst centre of infection ; the next is the improper triage and field hospital.

In regard to shock cases, I do not believe that they fall properly under the line of medical treatment ; this is a surgical proposition, and I believe the surgeon is better prepared to take care of this work and that he wants to do it.

I think the shock cases belong to the surgeon, and the medical men should recognize that this is true.

Lt.-Col. Lee. In the last offensive the hospital for seriously wounded men was located 18 kilometres south of the line ; for 36 hours we had no evacuation hospital behind us at all, no ambulances, no trucks. The field hospital handled 987 patients and operated 335 cases. No one deplores more than I the doing of surgery in field hospitals by inexperienced men, but if a capable surgeon could be placed to teach adaptable men to do surgery, good work could be done.

On one occasion our men worked 20 hours at a stretch, and there was no hospital available behind us. We have never done head cases or spine cases; these should go back. We have done abdomens and chests, and compound femurs in shock, also bad multiple wounds. If we were to have gone on for a number of months it would have been advisable to have a small mobile unit attached to the division.

General Finney. This is a very important point. In the 2nd Division they have put up a wonderfully good demonstration of what can be done in a field hospital. The only question in mind is whether this was an individual thing — whether it was because Colonel Lee and his unit were working together with the Division Surgeon that they did such splendid work, or whether it is a demonstration of what we all ought to be able to do.

At the present time I am not convinced that the same idea could be transplanted and found to be for the greatest good to the greatest number; it has worked there but it might not work as well elsewhere.

Colonel X... There is one point of danger in this scheme : that is that the field hospital will be converted into a triage hospital or a specialized hospital. It seems to me that our field hospitals should be trained to do everything possible — medical cases and surgical. There is danger that the gas hospital should do nothing but gas cases and the surgical hospital nothing but surgery.

Given a division that has a front of 30 or 40 kilometres, there will be 4 or 6 hospitals for different cases. Another sector of 10 to 12 kilometres will have one hospital, with a gas hospital acting also as triage. We cannot always pick cases and send them to specialized hospitals. With a division that is advancing, we should group hospitals and have them do the triage, but we should not train field hospitals along any one line. It makes no difference whether you call it a mobile, field, or evacuation ; all depends on the men and equipment. If there is a good surgeon at a field hospital, that hospital will do good surgery. I should prefer the field ambulance as the British have it ; our field hospitals should be *generally* trained, not *specially* trained only, and should be ready to do emergency work of any kind.

General Makins (British). I think the last speaker has touched the great point : military surgeons must be general practitioners. It is quite true that you may have special units for special work, but the surgeon ought to be ready to take anything that comes into his hands.

Colonel Cummins. I want to endorse the remarks made by the last two speakers. I feel quite certain that no divisional unit can avoid being ready to do anything that comes along ; it must be general and not special.

Certain points require special study. The best field ambulance I had had specialized in the immobilization of fractures, in the arrest of hemorrhage, in the feeding and warming of patients, in dealing with shock cases, and in triage and clinical work. I say clinical work because it is a most important matter to a divisional commander to know what becomes of the wounded and sick that come down the line. Those are the general subjects on which field ambulances ought to specialize.

I believe our field ambulance ought to be identical with your field hospital.

General Bowlby. I have listened to this discussion with the greatest interest, but I want to say that it is impossible to lay down hard and fast rules and regulations. Personal initiative is the thing to which a great deal must be left ; conditions vary with the times. What is right one time is wrong another time. While fighting is going on it is most important to pass people through as rapidly as possible to the rear. In cases where the line is fixed and the fighting not very heavy, it seems to me that the arrangements made in the British, French and American armies are very simple and good ; the difficulty comes in where there is a very heavy crowd of wounded.

Our feeling is that triage had better be done at the casualty clearing station. Don't let anyone worry the men and examine them too much; pass them on; give them to the casualty clearing station, or your evacuation hospital, and there let their fate be decided. Is a man to be retained for operation, or is he to be evacuated? The sooner you get the men to the evacuation hospital the better, and the heavier the fighting the more necessary it is to get the men there as quickly as possible. The object of the field ambulance is to have the patients dressed, examined, and passed on. There are cases where it is advisable to do operations there, but the point of view to keep in mind is that when you have operated a man you have not finished with him, and if you have no way of caring for him afterward it is better not to do anything; you are not doing him a kindness by operating him if you cannot give him after-care. It is necessary to have the man under favorable conditions and where he can be retained after operation. The field hospital may have to be moved and patients moved with it who are much better not moved.

In a small manual published for the guidance of our surgeons it was advocated that no operations should be done at the field ambulance except arrest of hemorrhage and removal of badly smashed limbs. This is essential. Under these circumstances you may require resuscitation teams, and we have put them lately in field ambulances to enable these operations to be done more thoroughly; but whenever a patient can be evacuated he should be sent to a casualty clearing station. It is essential to free the field ambulances and not block them.

On the 8th of August the 4th Army began a large move, and within three weeks we had moved our casualty clearing stations 34 times. There is an idea that an evacuation unit is a fixed unit, but it is not. If you say you must move the whole hospital, it becomes an impossibility; but you can take a section of the casualty clearing station with operating teams and a sufficient number of staff to deal with 300 to 400 patients and put them forward. Put them in the field ambulance and there they can do the work, and while they are working the field ambulance can move up. There is too much of a habit of talking of the C.C.S. as an indivisible unit; some of the surgeons can be moved on to aid the staff of the field ambulance. If you supply operators from the C. C. S. and re-establish the C. C. S. in detail — not all at once, the thing is not impossible.

One of the greatest difficulties in our advance was the transportation of the C. C. S. forward, or bringing patients back. Our chief difficulty came from the number of canals with bridges blown up. The C. C. S. was often ready to go but had no opportunity of moving on because of the pushing-up of military material. But when we could not move it as a whole we could move sections of it and re-establish it on the other side of the canal. If we look at other parts of the world where the war has been going on, we will find other difficulties which we have not encountered. Our conditions were entirely different from those of the Italians in mountainous regions.

The general principle is to get wounded back from the forward areas as soon as you can. Do not operate unless you can look after the cases subsequent to operation. If you insist on operating a large number of cases, many of whom will have to be moved, you will lose many lives.

Colonel Crile. **Shall Evacuation Hospitals be specialized hospitals or not?**

General Wallace. As far as the front area goes, I think that on the whole every clearing station should be equipped in the same way to deal with every class of case. I quite appreciate the benefit of having hospitals for head cases, abdominal cases, etc., but this means great care in arrangements that the staff at the abdominal hospital has got adequate work; the same thing at the chest hospital, etc. In a battle you can never prognosticate the proportion of different cases which you have; consequently, if you separate them, you are apt to get one hospital very hard worked while another has nothing to do at all. This is the surgical point of view. From the administrative point of view, the difficulties of evacuation in the front area are extremely great, and I don't think it is fair under those circumstances to put an extra burden on people who are already overworked. It is much better, therefore, to equip hospitals so that they can deal with every class of case and let evacuation go along on quiet lines, but from what I see I really think that at the base there is great scope for specialization, for there you can always assure an even flow of cases, and the personnel is always fully equipped.

Lieut.-Colonel Cannon. The disadvantage that comes from specialized hospitals was demonstrated at St. Mihiel. There, one hospital was set aside for very badly wounded, that was not farther away than the other hospitals but was quite separate from them, with the result that no provision was made in the way of donors for blood transfusion. Blood was badly needed, and there were no slightly wounded or gassed men from whom blood could be obtained for these cases.

General Wallace. It is not really a question of specialization, but a question of separating walking wounded from more seriously wounded. In the British Army we put aside one station for walking wounded ; they can be dealt with by a small staff and quickly sent away. From a surgical point of view the great object is for a hospital with seriously wounded not to have too much work. One can't help being struck by the air of calm in a station dealing only with serious cases.

Colonel Crile. **Is it advisable to establish a pool of medical officers and nurses to supply emergency personnel when needed, and to render possible the utilization of all personnel at all times ?**

General Wallace. Up to quite recently the fighting has only really involved one or two armies, and our armies were asked : " What is the minimum amount of personnel that you can do with, if the army remains quiet ? " Directly one or two armies got involved, the Medical Department knew what personnel was needed. Surgical teams were supplied and were moved into the fighting area. There was no definite pool, but they knew what other armies could spare.

GENERAL BOWLBY. I think the experience we had at the front is that when everything is quiet there is no objection to establishing special hospitals, but when operations get active we can no longer separate head and lung cases, and every C. C. S. must be prepared to take, in heavy fighting, everything that comes along. In times of quiet it may be distinctly advantageous to the patients to have specialized hospitals.

COLONEL CRILE. **What is included among non-transportable cases? Suppose we say cases of hemorrhage, shock, wounds of the chest, as a beginning, what other cases should be included?**

GENERAL BOWLBY. I think all completely smashed limbs should never be removed from the field ambulance.

COLONEL CRILE. **If there is a sufficient number of hospitals shall one mobile hospital be set aside for the care of the walking wounded?**

LT.-COLONEL JOHNSON. I think the mobile hospital is such a valuable unit that it should not be used for this work; it should be put to the use for which it was intended, and not used for walking wounded.

GENERAL BOWLBY. In the recent advance of the 1st Army there was a walking wounded hospital and three others. I think it very desirable to have a walking wounded hospital in the same direction as, or beside the other hospitals. It enables a string of ambulances to go in the same direction, and a given ambulance car might deposit three walking wounded at one place, and other patients at another place. I remember seeing a walking wounded officer with a small wound on the outer side of his thigh. I was asked to see him because of a swelling, and I found that he should become a lying patient. To distinguish between walking and lying wounded you need extremely competent surgeons. Out of 20 walking wounded, usually at least one ought to be taken to the hospital which deals with other cases. Often a large number of soldiers will crowd on to a light railway or van in order to get away, and if three or four lorries carry away walking wounded, men will get on who should be considered as lying wounded. In case of crowding, a hospital for walking wounded should have a train attached to take away the men as soon as possible and relieve the C. C. S. Of course the patient should not be taken a great distance. A large number of walking wounded are always the first to arrive, and they occupy the time and the operating theaters in such a way that when the seriously wounded arrive a large number of cases are in front of them. If walking wounded can be put in a particular train and sent to another area, you relieve your own staff and bring into work a large number of officers at base hospitals who otherwise would have nothing to do. With large numbers of wounded, we have endeavored to have temporary ambulance trains and pass on the walking wounded to the base, where they will arrive soon enough for their wounds to be carefully treated. I think that when making provision for walking wounded you should also make provision for special cars.

All cases of fractures should be sent to regular hospitals for treatment, and temporary ambulances should pass them on. You then bring into action a large number of medical bases, as well as hospitals at the front, and therefore deal with a much larger number of patients.

COLONEL CRILE. **Should mobile and evacuation hospitals be grouped in threes, in fours, or should they be isolated ?**

COLONEL REYNOLDS. Group all hospitals where possible.

COLONEL LYLE. From a transportation point of view, I agree. Group all hospitals as near together as possible.

COLONEL CRILE. **Is it advisable to establish a rotation of service between the personnel of advance and rear units ?**

GENERAL FINNEY. It seems to me that it would be to the mutual advantage of every one concerned — those working in the forward areas, in base areas, in intermediate areas — if each knew something at least of the problems of the others. There would then be less tendency to criticise, to feel that the other fellow who had seen the case first was not quite on to his job. I find that the more a man knows other problems, the more charitably inclined he is. Therefore it is desirable that we should institute some interchange of officers from base to front. After consultation with the administrative authorities, it was decided to change at the rate of 10 o/o a month the personnel of base and front areas. That would take about ten months to bring about a complete change of personnel.

Perhaps it would not be desirable to have a complete change; it might not be possible; but it seemed eminently desirable to have a considerable change of personnel from the front to the rear and from the rear to the front. This scheme was being worked out when the war closed. I think it has many desirable features. I think a man really can do better work at the base when he has had front line experience; nothing can bring out a man more than front line work. I feel sure that for the morale of our profession, it would be to the advantage of every one concerned to say nothing of the care of the patient. The idea was to keep a continuous circulation of personnel. Whether it would work or not I don't know; we had no opportunity to try it. I myself believe that it would have worked satisfactorily.

General Makins. I am very glad to hear General Finney raise that point. I have had large experience at base hospitals and I have always felt that it was desirable to have changes made. The most important reason is that it gives the individual medical officer a proper idea of the course of a surgical case. The majority of the men who have worked at one stage of the line have gained no experience of military surgery whatever. I have always felt that it was quite possible to have a definite arrangement by which a certain number of the men could move.

The other point that I have felt so very strongly upon is that when the men are moved they should not really interfere very seriously with the composition of the staff for the time being. The system with us was for new men who came out to be assigned to a base hospital, which served as a depot to supply medical officers at the front. I believe that is an extremely bad system, as these men interrupt the work of the hospital because they are not used to it, and in a few days they are shifted to the front.

I believe there is one method which would make a very great difference. At a base hospital, you get two classes of cases: serious ones, and patients who stay a very short time, or slight cases. I think that all large base hospitals should be divided in two classes: 1. Serious cases, with a staff as permanent as possible. 2. Class of casualty clearing station. This is a very important point economically and medically. There is no harm in the latter hospital in changing the staff often; you can look after the patients without interfering with the work.

Economically it is a very extravagant method to pass men from base hospitals into regular hospitals. They must have everything provided for them, and some arrangement could be made by which hospitals of two classes could be a great advantage to the medical service with due economy. The idea of letting men see the progress of a case from beginning to end is very good, and it engenders confidence of one set of surgeons in the other. It is often felt that the place of a medical officer at the base is not so important as that of the medical officer at the front. This is a mistake. When a patient goes home and a verdict is passed on his case, it is never passed on the officers in the front line; they are scot free of criticism. If a man is supposed to have been badly treated, the medical officer at the base is criticised, not the surgeon at the front.

Colonel Lyle. There is the question of transportation. It is difficult to provide the necessary transportation.

Colonel Crile. **What is the best method to prevent introduction of infectious diseases into first line organizations by replacements?**

General Sir John Rose Bradford. It seems to me that the only method of doing that is to have some organization segregated for that definite purpose. At present, all cases of infectious diseases are sent to a field ambulance.

Colonel Cummins. First of all one has to examine the means by which replacements are made. We have a base depot at which the troops join; from there they go to the divisional replacement battalion. We have on the other hand a corps depot through which replacements for the corps go, and in some cases a divisional unit of the same kind.

All depends on close inspection. In time of disease this would be done more carefully than usual. Certain things always escape us. It is quite certain that it is impractical to suggest swabbing of throats of the men in the depot to make sure that there are no carriers. A very important point is to have a medical officer make the inspection and pick out all men who look ill and examine them. Both at the base depot, divisional replacement units and corps replacements units, there must be a few tents put aside for the immediate isolation of any one suspicious. There must be close touch between the medical officer in charge of these units and the nearest mobile hospital.

My scheme would be to have an empty isolation hut for suspected cases, an organization to separate and observe contagious cases with good sound routine and inspections, but no elaborate system.

### PROBLEMS RELATING TO THE CARE OF PATIENTS.

COLONEL CRILE. **Has our experience in the Argonne brought out any new points? Has anything new developed in the treatment of shock and hemorrhage?**

LT.-COLONEL CANNON. I have had cases reported of men being brought back on stretchers with blankets over them and not under them. These men lose heat by sweat and by their wet clothing. They lose it by contact with stretchers which may be wet. It is very important to conserve the heat of the patients and see that they are properly blanketed. Report came in during the recent activity that patients in shock had four blankets over them and none under them.

I have sometimes urged that shock teams be sent to the dressing station because of the time element and low blood pressure. It is desirable that shock teams be got to work as early as possible. As a matter of fact, I think shock teams have not been working so far forward in most places.

COLONEL CRILE. **What can be done for shock during the journey in the ambulance?**

GENERAL WALLACE. The heating of our ambulances is done by a system of running tube pipes under the seats and into the exhaust, but the exhaust is prevented from getting into the ambulance. It has been satisfactory on the whole, but sometimes the ambulance gets too hot, which is bad for wounded men.

LT.-COLONEL CANNON. We had an accident in our army which seemed to determine our action in this respect. A pipe burst, the gas leaked into the ambulance, and the patient were almost asphyxiated. We might have a pipe running from the hot water system to the engine, when there would be no possibility of this accident occurring. Something of this sort seems desirable along with the provision of hot water bottles in case the heating of the ambulance is not good or is lacking.

GENERAL WALLACE. We have had no question of poisoning but I would prefer to bring the pipe underneath where the stretcher lies.

LT.-COLONEL CANNON. Another question is as to the arrangement or caring for these patients in mobile and evacuation hospitals.

and whether it is really proper to have teams constituted of medical men rather than surgeons. For future policy it seems very desirable to have some opinion on that point.

General Bowlby. The question of warming patients comprises warm cars, warm admission rooms, and warm operating rooms. You must see that the men do not get cold in the operating theater. It is a good idea to put a small stove under each operating table so that the patient is kept thoroughly warm during the operation. Patients have often been kept warm until they got to the operating theater and not warm enough during the operation. A small stove burning under the table does a great deal to prevent patients getting chilled.

Colonel Crile. **What during operation?**

Captain Middleton. The question of warmth supplied by sandbags heated in the old-fashioned way will relieve the question of heat under the table.

The question of donors for evacuation hospitals could be solved if we could have co-operation : if one case of slightly wounded was brought in to each five or eight cases of seriously wounded. In that way the unit has a constant supply of donors.

Colonel Crile. This is a very valuable discussion. I wish to interrupt the program for a moment and say that the A. E. F. is fortunate in having as a part of its surgical personnel Lt.-Col. Cannon who has done notable work. I will call upon him to speak of his work at the laboratories at Dijon.

Lt.-Colonel Cannon. There are certain things that have been brought out already as important matters in the treatment of shock, and which have been long recognized. Among the first is the question of heat. It has already been pointed out that a man suffering from a severe wound is likely to lose heat because of sweat and wet clothing. Another cause is low blood pressure, and there may be a reduction of 50 o/o in heat production; therefore the patient has a tendency to become cold and get into a worse condition surgically. Every effort should be made to keep up the heat of the man in all stages, from the time of wounding until recovery.

There is danger in over-heating because he is likely to have lost fluids by bleeding or sweating. If you over-heat him he will lose more fluid because of sweating.

In addition to the loss of body heat, and the necessity of keeping up body heat, there are several other points. A characteristic of a shocked man is that he has low blood pressure. However we may differ regarding the cause of shock, whether we regard it as primarily a matter of disturbance of the nervous system or as being caused by chemical causes, there is this consequence : the low blood pressure that prevails in the shocked man and which is likely to cause damage to the nervous system, because low pressure means slow circulation and with slow circulation there is a lack of oxygen in the tissues, which makes them likely to suffer.

When the blood pressure is lowered by shock there is a critical level in the fall of the blood pressure, below which it becomes inadequate to keep a sufficiently rapid circulation to supply the tissues with enough oxygen to keep them normal. If this inadequate oxygen supply is allowed to continue there is damage done to the organism which gets greater as long as the condition persists. If you allow a man who is in shock or who has had a severe hemorrhage to persist in a stage of low blood pressure, he suffers damage from which it is often impossible to recover. That is the reason why the operations of the resuscitation teams should be begun as far forward as possible.

The question is asked as to what should be done during operation. If a man has suffered shock and had damage done to his central nervous system, he is extremely susceptible to ether and chloroform anesthesia. In a series of cases I worked on under General Wallace's auspices and help, we found that the average fall of blood pressure during operation was from 88 down to 62 in the course of operation. When a man has already had a low blood pressure or is suffering from low pressure, and you seriously lower the pressure still further, it is quite obvious that he is liable to undergo serious damage in consequence of that drop. If you have to use ether in these cases, a favorable condition is to make use of transfusion or other means of raising blood pressure during operation. If possible, use nitrous oxide and oxygen which gives exactly the same degree of anesthesia without diminishing the blood pressure, but it must be used in the ratio which gives the greatest amount of oxygen. If you use too much nitrous oxide you get lowered blood pressure as with ether. The ratio should be 3 to 1.

I should say, therefore, that we have first of all :

1. Warmth as a cardinal point of treatment.

2. Prevention of prolonged persistance of pressure below a critical level.

3. Rest, both because of the effects produced on the nervous system and injury, because every movement a man makes requires oxygen, and with inadequate oxygen supply it is necessary to reduce the demand for oxygen.

4. Fluid to replace loss during shock, especially if attended by hemorrhage.

PROFESSOR BARCROFT. I entirely support what Col. Cannon has said regarding loss of oxidation in low blood pressure.

COLONEL CRILE. We will resume the practical side. As a basis for discussion let us say that the treatment for shock is:

1. Warmth.
2. Rest and sleep.
3. Fluids.
4. Morphia.
5. Transfusion and various methods of raising blood pressure.

COLONEL CRILE. **Is morphia contra-indicated in abdominal perforations? In chests?**

GENERAL WALLACE. I expressed my own opinion a long time ago. I don't think it right for any benefits to be got in the diagnosis to deny any man an adequate dose of morphia. I certainly always advocate it, and I do not think it complicates the diagnosis. Even if it takes away a man's sensibility, which I don't think it does, one has enough faith in one's self to operate if there are chances of recovery. In large doses morphia is bad.

MAJOR CASTELLANI (Italy). I quite agree with General Wallace. Morphia is most useful.

LT.-COLONEL YATES. Out of 130 chest cases, I have seen two in which morphia seemed to have a bad effect, and I am inclined to think it was not due to the fact that it had been given, but because the patients had been morphinized before and no record made on their cards. Therefore, I think there is no objection to giving morphia judiciously in chest cases.

COLONEL CRILE. **In battle conditions, are the tissues of the wounded desiccated? Is water of more value to the organism if it is absorbed through mucous membranes; from the subcutaneous tissues than when given intravenously? That is, should water have a biological pass?**

PROFESSOR BARCROFT. I have something to ask. In gassed patients suffering from shock we had been advised to give large quantities of hot coffee on account of the caffeine. I have wondered if it was not the water that was beneficial instead of the caffeine?

CAPTAIN ROBERTSON. I have seen men coming to a base hospital 24 hours to a week after a hemorrhage, and when we made a test of blood volume at that time we found it much reduced, even days after a primary hemorrhage, showing that circulation had not been restored. I have seen as much as 60 o/o reduction after a week. What was it due to? It is ordinarily known that after a hemorrhage the body tends to make up the loss by pouring fluids into the circulation. In these cases it had not made up the volume because the tissues had been desiccated and had no fluid in reserve. We began by giving these men large quantities of fluid : water by the mouth, and saline solution by the rectum. We gave as much as 5000 cc. The blood volume was quickly restored to normal. We found that these patients could take an astonishing amount of fluid by rectum. With the rise in volume there was a rise in blood pressure.

By measuring the intake of fluid and the output of urine, which normally is 3 to 2 (3 of intake to 2 of output), we found that some of these patients took very large quantities of water for relatively small quantities of urine, — 5 to 6000 cc. of fluid and only 6 to 700 cc. of urine.

A case came in with a blood volume of a little over 3000 cc. In 20 hours the man took 5000 cc. of fluid and the blood volume increased to 4700 cc., the output of urine being only 700 cc., showing that the fluid was taken both by the circulation and the tissues. Conditions at the battle front were favorable for a drying-out of the tissues. The men in line have very little to drink; their fluid intake is very small; they work hard; they sweat a lot; and they drink little. When they are wounded they sweat and have hemorrhage. If a man has a hemorrhage when his tissues are dried out he stands it worse than when he has had enough fluid.

GENERAL BOWLBY. I think there is a great tendency to put the fluid directly in the man's veins instead of in his stomach. I have been asking lately if the man has had enough to drink. I believe that almost all these men who are admitted in a state of shock should have large quantities to drink, if they are able to drink. If they are unable to drink the next best is to give it them by the rectum. Very large quantities of fluids are absorbed by the rectum, and in many cases it does more good to give pint after pint through the rectum than one pint through the veins. The natural method of absorption will retain the fluids and utilize them better than if they are put into the blood, hence the necessity for giving the patient plenty to drink.

COLONEL CRILE. **How much injury to the donor is the taking of blood for transfusion? How many days' disability for duty should a man have?**

CAPTAIN ROBERTSON. There is very little harm done from bleeding. The average amount of blood needed for transfusion is 500 to 600 cc., and the average man stands that very well. If he lies down one or two hours he can go about his work without feeling any difference. The only instances when a donor is disturbed is when he has been in line three or four days with little sleep, when he has suffered from exposure, and been hungry and thirsty.

COLONEL CRILE. **How long may citrated blood be kept before using?**

CAPTAIN ROBERTSON. I don't think it possible to say how long citrated blood may be kept before being used; it depends a great deal on the technic in which the blood was drawn. With good technic, very little change takes place and the blood may be kept some time, practically twenty-four hours, or even longer. If the inflow in the citrator has been rather slow and the transfusion not done very well, the blood will have undergone a change and you may get reaction. The aim in transfusion is to get the blood in the recipient as soon as possible. There is no objection to keeping it several hours.

In regard to the use of preserved blood for transfusion I will say a word or two. I have kept blood as long as 25 to 26 days, and the transfusion had the same effect and was just as good as with fresh blood. In using this preserved blood there were several conditions which made the method seem quite practical. Warfare was then pretty stable; hospitals were pretty well established and attacks were local. During such attacks large numbers of wounded came to the C. C. S., and for two or three days we would have a tremendous flood of wounded. The resuscitation ward was filled and it was impossible to give transfusion by the ordinary method. If you can store blood beforehand you can give a larger number of transfusions in the same time. Under those circumstances this method worked out very well, and we gave a great number of transfusions.

In the last three or four months our hospital became mobile and this method was not of as much use. It was too much trouble to move a large quantity of bottles, etc., and it was easier to transfuse by the usual method. If there was any difficulty in getting donors, the blood could be taken at a central place and distributed. However, it seems more practical to regulate the donor supply.

COLONEL CRILE. **Are salines as good as blood? As good as gum salt? And in what class of cases, if at all, should gum salt be used instead of blood?**

MAJOR MIXTER. I have seen two cases in which gum salt did harm. One was a case of hemorrhage which had not been held for resuscitation at the dressing station, and while waiting for blood was given a transfusion of gum salt. The man died within half an hour. The other case was similar. These deaths were apparently caused or at least hastened by the gum salt.

LIEUT.-COLONEL WOLSEY. I have inquired of many evacuation and mobile hospitals what their opinion was of gum salt solution. In no single case did I get a favorable opinion.

X... X.... Without any hesitancy I should say that gum salt solution is absolutely contra-indicated. Blood pressure is increased sometimes from 60 to 130, but almost invariably it drops to 40 or 50 after a short time.

LIEUT.-COLONEL LEE. We have had one very excellent result from the use of gum salt; the patient was a man in a serious condition of shock after hemorrhage. The result following the introduction of gum salt was very remarkable and the man made a very nice recovery. On the other hand two cases in a similar condition collapsed after the use of gum salt.

CAPTAIN MIDDLETON. We have had two experiences with gum salt. The results on patients received very early — 3, 8 or 12 hours after being wounded — were uniformly good. In a second series of patients who were received 3 or 4 days after being wounded, the results were universally poor. The difference in results could be attributed to the difference in transportation, to exposure, and loss of body heat and fluids in the second series of cases.

At Mobile Hospital No. 1, we proceeded to give citrated blood as a uniform method of resuscitation, and our results were just as disappointing with citrated blood as with gum salt. Out of 13 cases of transfusion in 3 days, 8 died and only 3 showed a normal effect.

I should say that the results that have been obtained lately are due not to inferiority of methods, but to the fact that you are dealing with conditions entirely different from those of summer months. In winter we are applying methods of resuscitation near the front.

Captain Robertson. I was asked to look into the method of giving gum solution. I paid a visit to front area hospitals and talked with a large number of men and got various opinions. Some of the workers were enthusiastic and had got good results, some were luke-warm, and some were against it. On looking over the records it seemed to me that the poor results reported from gum solution were largely due to the choice of unsuitable cases. There are about 4 classes of cases from which good results cannot be expected :

1. Cases of shock for a long period — 15 to 20 hours or more, with so much damage done the tissues and brain cells that blood pressure is very low and transfusion has no effect.

2. Cases treated immediately by gum salt; patients brought into the resuscitation ward and not given time to pick up with heat, morphia and fluids. These do badly.

3. Cases suffering from very severe blood loss. Gum salt solution gives a temporary rise in pressure, but they have too little oxygen and the pressure is not maintained.

4. Gas bacillus cases. We made a postmortem of cases that did not do well after gum injection, and in every case we found gas bacillus.

Where these various considerations are taken into account and gum solution used on that basis, results are good.

Sir Walter Fletcher. It is a matter of great importance that the experience of the past months should be placed on record; I hope records have been collected. It is very important to have the clinical condition of the patients, the method of making the solution, and the method used for transfusion. There is certainly a wide variation in the quality used in France. In Italy, experiences have been uniformly unfavorable, because the solutions used were unsuitably prepared. Improvement in the solution changed the experience altogether.

Last week we received reports from Macedonia; the experience was bad, and it is clear that they had been using solution in the same form as in Italy.

Alkaline solution of gum is very difficult to prepare; great attention should be paid to filtering.

The point is not clear whether gum solution should be used; we have not yet received reports with enough detail to see if it should be used or not.

General Finney. In going round various hospitals I was struck with one point. In several of the hospitals I was told that the solution from the Central Laboratory was not giving satisfactory results. Solution made fresh in the hospitals gave good results. This happened in several hospitals. The reason for the unsatisfactory results was the question of time ; fresh solution gave good results.

Lieut.-Colonel Cannon. There is evidence on both points that Sir Walter Fletcher has brought up. The first one was the clinical condition of the patient at the time the solution was used. I have had experience under two conditions. When the patient was received early, the result was good. When the patient was received late the result was unfavorable. In one sector of the British Army last spring in an advanced station excellent results were obtained from gum solution. 18 miles behind the front, with the same method, unfavorable results were obtained. So the clinical condition of the patient is one that will very largely determine the value of the method. I have a letter from one of the resuscitation officers who reports that in transfusion of blood he got chills in cases of low blood pressure from shock.

There are differences in the gum used. The gum which has been used in the British Army was provided in clear lumps. In France, we had to get powdered gum and we found that it contained starch and..... I have removed 60 o/o blood volume from an animal and given it gum salt solution; the animal recovered. So both conditions mentioned are important.

From the very beginning I have urged that this solution be used as far forward as possible. Farther back I have found that in mobile units gum salt solution has been used instead of blood, because of the lack of donors, with bad results.

If an organism has been suffering from low blood pressure for some length of time, it makes no difference whether you introduce blood or gum, you get unsatisfactory results. I would emphasize that it is the first essential to have these resuscitation measures applied as soon as possible, before damage due to low blood pressure has come to a point from which the patient cannot recover. I would like to know whether persons who have used salt solution and think favorably of it have made blood pressure observations to prove that it does any good whatever. I believe that if you introduce salt solution you get a rise in the pressure, but I do not believe that it is permanent. Gum salt remains in the blood vessels and salt does not.

Last Friday, at a meeting in Boulogne of men who had had experience in the British Army, the following statement was approved by the committee.

"1. Provided that the gum solution is prepared from good gum, with a raised body temperature, and slowly injected, no seriously harmful results need be apprehended in its use.

2. The amount injected should be 700 cc."

COLONEL CRILE. **In emergencies, may grouping be disregarded?**

Does any one object to answering "Yes"?

COLONEL CRILE. **In the anemia by a tourniquet, are damaging chemical compounds formed?**

LIEUT.-COLONEL CANNON. Shock can be produced by shutting off the blood circulation for a time. We have had a number of cases of men who have had a tourniquet on for some time after being wounded and who have gone in shock on removal.

COLONEL CRILE. **Is the blood of a gassed case as useful in transfusion as the blood from a normal one?**

X... X... (French). In the French Army we use the blood of gassed cases for transfusion. What could oblige us to refuse transfusion from a gassed case? We may be sure, from experience, that there is no poison in the blood. Of course one will not use as a donor a man who is in an acute stage of gas poisoning, but I don't see why we should not take blood for the making of citrated blood from slight cases. There are many men in favor of keeping this blood, because it seems to be the best blood for transfusion, because it is concentrated with a high percentage of red cells, and is very apt to take up oxygen.

LIEUT.-COLONEL CANNON. I made use of blood from slightly gassed cases in July last, and called attention to the availability of these men as donors. There is no harmful effect but definite value to be got from this blood.

COLONEL CRILE. **What is the anesthetic of choice? What is the field of local anesthesia? Of spinal?**

LIEUT.-COLONEL CLINTON. The American Army is obliged to use ether almost universally. A few operators have facilities for using nitrous oxide, but the standard has been ether through necessity. Nitrous oxide is preferred if available.

General Wallace. We have found gas and oxygen by far the best anesthetic in cases of shock. The worse a patient is, the easier it is to operate him by gas and oxygen, and it is the safest to give him. Apart from that, the usual anesthetic in the British Army is warm ether. We have avoided chloroform as a general anesthetic except for the purpose of conduction; still for a limited number of mouth and chest cases it can be used combined with oxygen.

Spinal anesthesia has not been much used; it is not advisable to use it if one is not thoroughly educated in its use.

Local anesthesia has been used in conjunction with gas and oxygen in particular cases; it is very useful in abdominal cases; it has been used by those who have been skilled in its use, and good results have been obtained. Generally local anesthesia takes too long to act.

We feel under a great debt of obligation to the American Army for their skilled anesthetists, especially in gas and oxygen. Gas and oxygen, as it has been given by skilled people, has saved many lives of patients under shock.

Colonel Crile asked General Makins to comment on the influence of war surgery on the surgeon when he returns to civilian practice.

General Makins. **What effect will military surgery have upon civilian work at home?** It cannot be thought that it will have any marked influence on surgery at home. In civil life we should always open an abdomen, and in certain cases get successful results as in the army. It is only in this war that facilities have been provided for taking the cases early enough. One thing in civil surgery which has been developed is surgery of the chest. When we come to surgery of the lung I think there is no doubt that there will be more of that; still, those who have only gained experience in military surgery will find lung surgery very different in civil life. As to the question of the development of the surgeon, it is quite clear that the work of a military surgeon must develop many qualities in a man; it develops quickness in making up one's mind, resourcefulness in meeting many great difficulties, etc. There is, however, one side where military surgery is not good as an example for the civilian surgeon; the personal responsibility is much greater in civil life. Younger men will have to bear that in mind when they go home and operate.

Colonel Crile. The program is now completed and there remain for me two pleasant duties to perform. The first is to express, though inadequately, our appreciation of the part taken in this meeting by our British, French and Italian colleagues, who have given freely of their wider experience in elucidating our problems.

My second duty is to say something on our own behalf. I have had opportunity in the course of my duties to see the work at many mobile, evacuation, and base hospitals. In our first offensives, through lack of experience in military surgery on the part of some of our surgeons in certain instances, the work showed deficiency, but in the last offensive the work was uniformly of a high order. In view of the progress made during our brief experience, I feel that we have reason to be highly gratified with the results secured.

